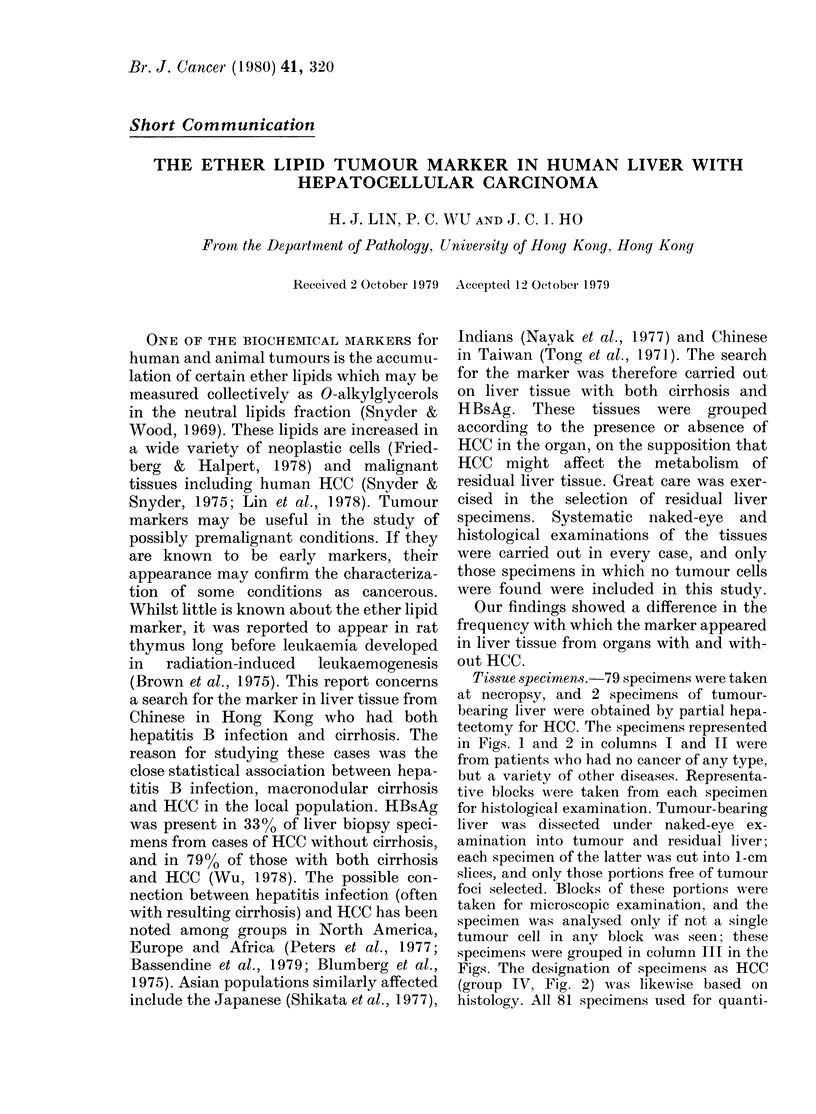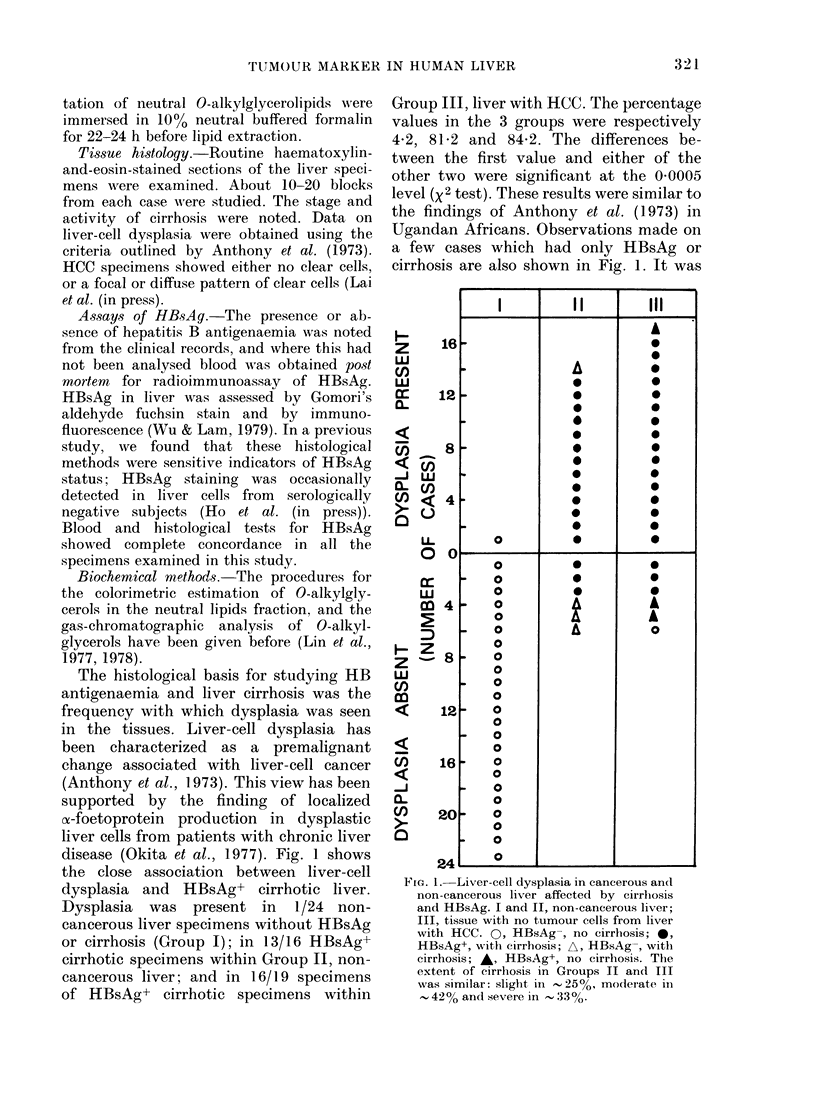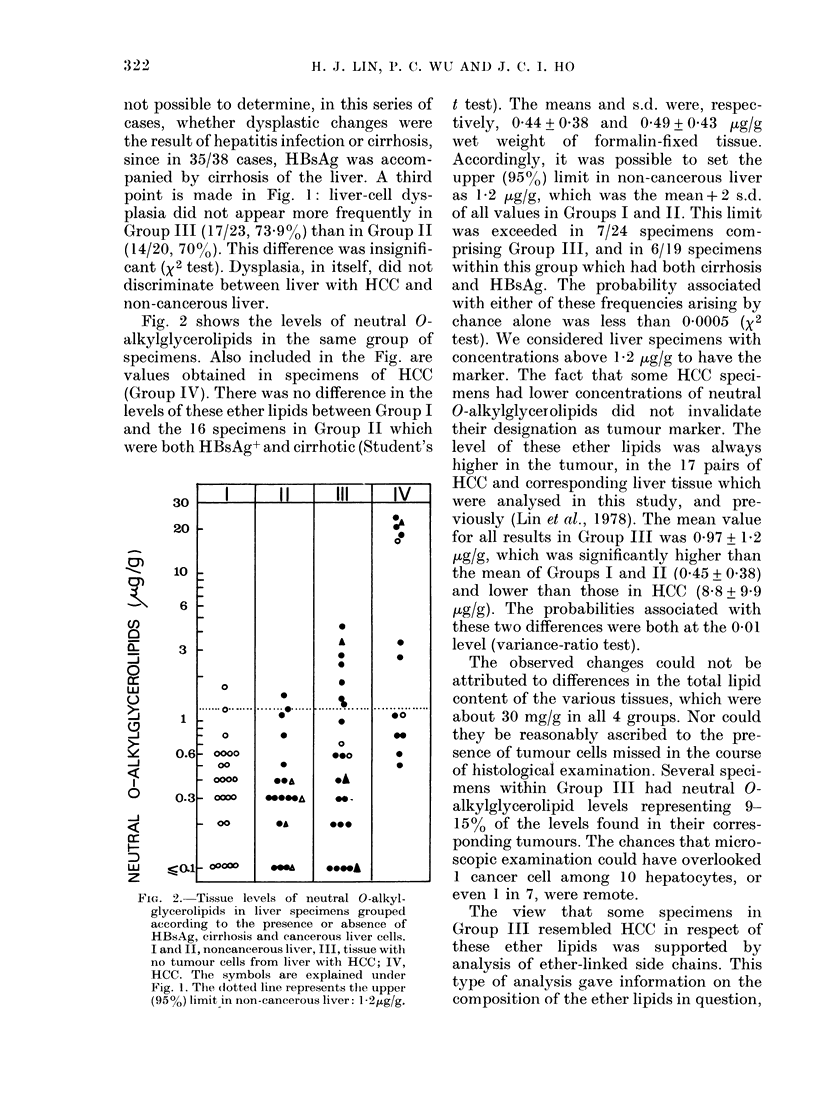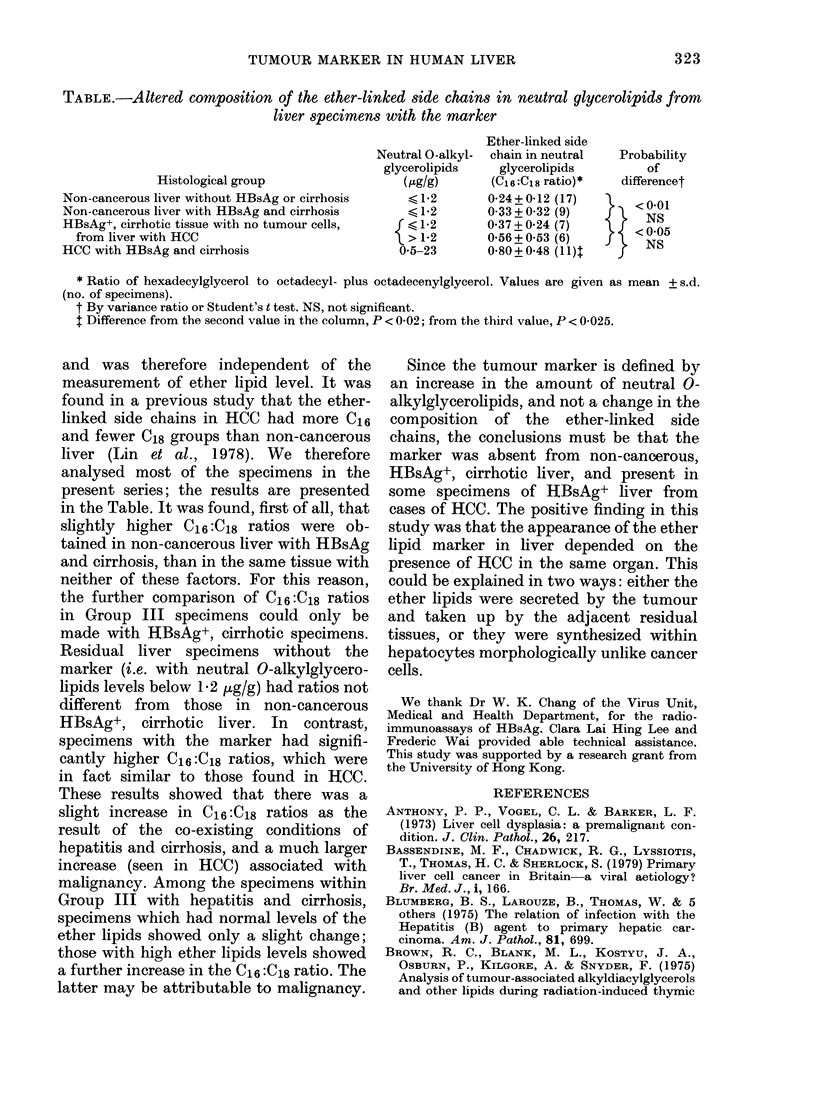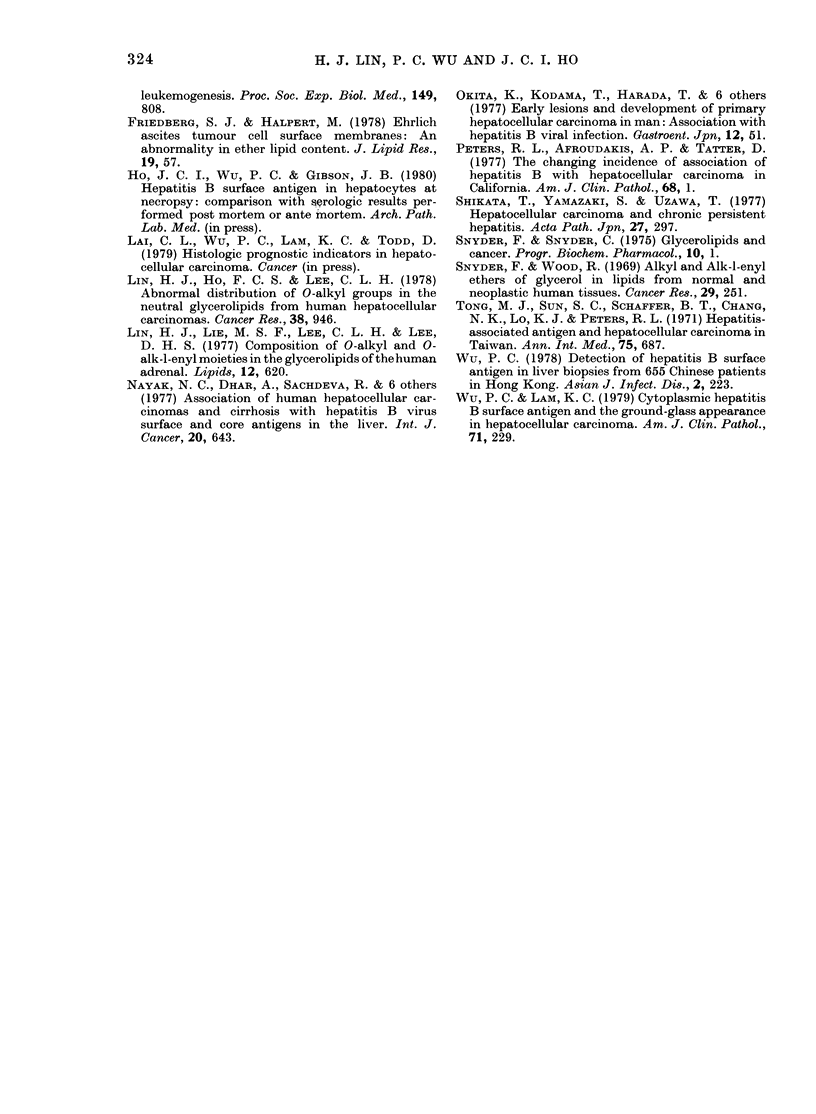# The ether lipid tumour marker in human liver with hepatocellular carcinoma.

**DOI:** 10.1038/bjc.1980.48

**Published:** 1980-02

**Authors:** H. J. Lin, P. C. Wu, J. C. Ho


*Br. J. Cancer* (1980) **41**, 320

### *Short Communication*

### THE ETHER LIPID TUMOUR MARKER IN HUMAN LIVER WITH HEPATOCELLULAR CARCINOMA

H. J. LIN, P. C. WU AND J. C. I. HO

*From the Department of Pathology, University of Hong Kong, Hong Kong*

Received 2 October 1979 Accepted 12 October 1979

ONE OF THE BIOCHEMICAL MARKERS for human and animal tumours is the accumulation of certain ether lipids which may be measured collectively as *O*-alkylglycerols in the neutral lipids fraction (Snyder & Wood, 1969). These lipids are increased in a wide variety of neoplastic cells (Friedberg & Halpert, 1978) and malignant tissues including human HCC (Snyder & Snyder, 1975; Lin *et al.*, 1978). Tumour markers may be useful in the study of possibly premalignant conditions. If they are known to be early markers, their appearance may confirm the characterization of some conditions as cancerous. Whilst little is known about the ether lipid marker, it was reported to appear in rat thymus long before leukaemia developed in radiation-induced leukaemogenesis (Brown *et al.*, 1975). This report concerns a search for the marker in liver tissue from Chinese in Hong Kong who had both hepatitis B infection and cirrhosis. The reason for studying these cases was the close statistical association between hepatitis B infection, macronodular cirrhosis and HCC in the local population. HBsAg was present in 33% of liver biopsy specimens from cases of HCC without cirrhosis, and in 79% of those with both cirrhosis and HCC (Wu, 1978). The possible connection between hepatitis infection (often with resulting cirrhosis) and HCC has been noted among groups in North America, Europe and Africa (Peters *et al.*, 1977; Bassendine *et al.*, 1979; Blumberg *et al.*, 1975). Asian populations similarly affected include the Japanese (Shikata *et al.*, 1977), Indians (Nayak *et al.*, 1977) and Chinese in Taiwan (Tong *et al.*, 1971). The search for the marker was therefore carried out on liver tissue with both cirrhosis and HBsAg. These tissues were grouped according to the presence or absence of HCC in the organ, on the supposition that HCC might affect the metabolism of residual liver tissue. Great care was exercised in the selection of residual liver specimens. Systematic naked-eye and histological examinations of the tissues were carried out in every case, and only those specimens in which no tumour cells were found were included in this study.

Our findings showed a difference in the frequency with which the marker appeared in liver tissue from organs with and without HCC.

*Tissue specimens.*—79 specimens were taken at necropsy, and 2 specimens of tumour-bearing liver were obtained by partial hepatectomy for HCC. The specimens represented in Figs. 1 and 2 in columns I and II were from patients who had no cancer of any type, but a variety of other diseases. Representative blocks were taken from each specimen for histological examination. Tumour-bearing liver was dissected under naked-eye examination into tumour and residual liver; each specimen of the latter was cut into 1-cm slices, and only those portions free of tumour foci selected. Blocks of these portions were taken for microscopic examination, and the specimen was analysed only if not a single tumour cell in any block was seen; these specimens were grouped in column III in the Figs. The designation of specimens as HCC (group IV, Fig. 2) was likewise based on histology. All 81 specimens used for quantitation of neutral *O*-alkylglycerolipids were immersed in 10% neutral buffered formalin for 22–24 h before lipid extraction.

*Tissue histology.*—Routine haematoxylin-and-eosin-stained sections of the liver specimens were examined. About 10–20 blocks from each case were studied. The stage and activity of cirrhosis were noted. Data on liver-cell dysplasia were obtained using the criteria outlined by Anthony *et al.* (1973). HCC specimens showed either no clear cells, or a focal or diffuse pattern of clear cells (Lai *et al.* (in press).

*Assays of HBsAg.*—The presence or absence of hepatitis B antigenaemia was noted from the clinical records, and where this had not been analysed blood was obtained *post mortem* for radioimmunoassay of HBsAg. HBsAg in liver was assessed by Gomori's aldehyde fuchsin stain and by immunofluorescence (Wu & Lam, 1979). In a previous study, we found that these histological methods were sensitive indicators of HBsAg status; HBsAg staining was occasionally detected in liver cells from serologically negative subjects (Ho *et al.* (in press)). Blood and histological tests for HBsAg showed complete concordance in all the specimens examined in this study.

*Biochemical methods.*—The procedures for the colorimetric estimation of *O*-alkylglycerols in the neutral lipids fraction, and the gas-chromatographic analysis of *O*-alkylglycerols have been given before (Lin *et al.*, 1977, 1978).

The histological basis for studying HB antigenaemia and liver cirrhosis was the frequency with which dysplasia was seen in the tissues. Liver-cell dysplasia has been characterized as a premalignant change associated with liver-cell cancer (Anthony *et al.*, 1973). This view has been supported by the finding of localized α-foetoprotein production in dysplastic liver cells from patients with chronic liver disease (Okita *et al.*, 1977). Fig. 1 shows the close association between liver-cell dysplasia and $HBsAg^+$ cirrhotic liver. Dysplasia was present in 1/24 non-cancerous liver specimens without HBsAg or cirrhosis (Group I); in 13/16 $HBsAg^+$ cirrhotic specimens within Group II, non-cancerous liver; and in 16/19 specimens of $HBsAg^+$ cirrhotic specimens within Group III, liver with HCC. The percentage values in the 3 groups were respectively 4·2, 81·2 and 84·2. The differences between the first value and either of the other two were significant at the 0·0005 level ($\chi^2$ test). These results were similar to the findings of Anthony *et al.* (1973) in Ugandan Africans. Observations made on a few cases which had only HBsAg or cirrhosis are also shown in Fig. 1. It was

FIG. 1.—Liver-cell dysplasia in cancerous and non-cancerous liver affected by cirrhosis and HBsAg. I and II, non-cancerous liver; III, tissue with no tumour cells from liver with HCC. ○, $HBsAg^-$, no cirrhosis; ●, $HBsAg^+$, with cirrhosis; △, $HBsAg^-$, with cirrhosis; ▲, $HBsAg^+$, no cirrhosis. The extent of cirrhosis in Groups II and III was similar: slight in ~25%, moderate in ~42% and severe in ~33%.

not possible to determine, in this series of cases, whether dysplastic changes were the result of hepatitis infection or cirrhosis, since in 35/38 cases, HBsAg was accompanied by cirrhosis of the liver. A third point is made in Fig. 1: liver-cell dysplasia did not appear more frequently in Group III (17/23, 73·9%) than in Group II (14/20, 70%). This difference was insignificant ($\chi^2$ test). Dysplasia, in itself, did not discriminate between liver with HCC and non-cancerous liver.

Fig. 2 shows the levels of neutral *O*-alkylglycerolipids in the same group of specimens. Also included in the Fig. are values obtained in specimens of HCC (Group IV). There was no difference in the levels of these ether lipids between Group I and the 16 specimens in Group II which were both $HBsAg^+$ and cirrhotic (Student's

FIG. 2.—Tissue levels of neutral *O*-alkylglycerolipids in liver specimens grouped according to the presence or absence of HBsAg, cirrhosis and cancerous liver cells. I and II, noncancerous liver, III, tissue with no tumour cells from liver with HCC; IV, HCC. The symbols are explained under Fig. 1. The dotted line represents the upper (95%) limit in non-cancerous liver: 1·2μg/g.

*t* test). The means and s.d. were, respectively, 0·44 ± 0·38 and 0·49 ± 0·43 μg/g wet weight of formalin-fixed tissue. Accordingly, it was possible to set the upper (95%) limit in non-cancerous liver as 1·2 μg/g, which was the mean + 2 s.d. of all values in Groups I and II. This limit was exceeded in 7/24 specimens comprising Group III, and in 6/19 specimens within this group which had both cirrhosis and HBsAg. The probability associated with either of these frequencies arising by chance alone was less than 0·0005 ($\chi^2$ test). We considered liver specimens with concentrations above 1·2 μg/g to have the marker. The fact that some HCC specimens had lower concentrations of neutral *O*-alkylglycerolipids did not invalidate their designation as tumour marker. The level of these ether lipids was always higher in the tumour, in the 17 pairs of HCC and corresponding liver tissue which were analysed in this study, and previously (Lin *et al.*, 1978). The mean value for all results in Group III was 0·97 ± 1·2 μg/g, which was significantly higher than the mean of Groups I and II (0·45 ± 0·38) and lower than those in HCC (8·8 ± 9·9 μg/g). The probabilities associated with these two differences were both at the 0·01 level (variance-ratio test).

The observed changes could not be attributed to differences in the total lipid content of the various tissues, which were about 30 mg/g in all 4 groups. Nor could they be reasonably ascribed to the presence of tumour cells missed in the course of histological examination. Several specimens within Group III had neutral *O*-alkylglycerolipid levels representing 9–15% of the levels found in their corresponding tumours. The chances that microscopic examination could have overlooked 1 cancer cell among 10 hepatocytes, or even 1 in 7, were remote.

The view that some specimens in Group III resembled HCC in respect of these ether lipids was supported by analysis of ether-linked side chains. This type of analysis gave information on the composition of the ether lipids in question,

TABLE.—*Altered composition of the ether-linked side chains in neutral glycerolipids from liver specimens with the marker*

| Histological group | Neutral O-alkyl-glycerolipids ($\mu$g/g) | Ether-linked side chain in neutral glycerolipids ($C_{16}$:$C_{18}$ ratio)* | Probability of difference† |
|---|---|---|---|
| Non-cancerous liver without HBsAg or cirrhosis | $\leqslant 1·2$ | $0·24 \pm 0·12$ (17) | $< 0·01$ |
| Non-cancerous liver with HBsAg and cirrhosis | $\leqslant 1·2$ | $0·33 \pm 0·32$ (9) | NS |
| HBsAg+, cirrhotic tissue with no tumour cells, from liver with HCC | $\leqslant 1·2$ | $0·37 \pm 0·24$ (7) | $< 0·05$ |
| | $> 1·2$ | $0·56 \pm 0·53$ (6) | NS |
| HCC with HBsAg and cirrhosis | 0·5–23 | $0·80 \pm 0·48$ (11)‡ | |

* Ratio of hexadecylglycerol to octadecyl- plus octadecenylglycerol. Values are given as mean $\pm$s.d. (no. of specimens).

† By variance ratio or Student's *t* test. NS, not significant.

‡ Difference from the second value in the column, $P < 0·02$; from the third value, $P < 0·025$.

and was therefore independent of the measurement of ether lipid level. It was found in a previous study that the ether-linked side chains in HCC had more $C_{16}$ and fewer $C_{18}$ groups than non-cancerous liver (Lin *et al.*, 1978). We therefore analysed most of the specimens in the present series; the results are presented in the Table. It was found, first of all, that slightly higher $C_{16}$:$C_{18}$ ratios were obtained in non-cancerous liver with HBsAg and cirrhosis, than in the same tissue with neither of these factors. For this reason, the further comparison of $C_{16}$:$C_{18}$ ratios in Group III specimens could only be made with HBsAg+, cirrhotic specimens. Residual liver specimens without the marker (*i.e.* with neutral *O*-alkylglycerolipids levels below 1·2 $\mu$g/g) had ratios not different from those in non-cancerous HBsAg+, cirrhotic liver. In contrast, specimens with the marker had significantly higher $C_{16}$:$C_{18}$ ratios, which were in fact similar to those found in HCC. These results showed that there was a slight increase in $C_{16}$:$C_{18}$ ratios as the result of the co-existing conditions of hepatitis and cirrhosis, and a much larger increase (seen in HCC) associated with malignancy. Among the specimens within Group III with hepatitis and cirrhosis, specimens which had normal levels of the ether lipids showed only a slight change; those with high ether lipids levels showed a further increase in the $C_{16}$:$C_{18}$ ratio. The latter may be attributable to malignancy.

Since the tumour marker is defined by an increase in the amount of neutral *O*-alkylglycerolipids, and not a change in the composition of the ether-linked side chains, the conclusions must be that the marker was absent from non-cancerous, HBsAg+, cirrhotic liver, and present in some specimens of HBsAg+ liver from cases of HCC. The positive finding in this study was that the appearance of the ether lipid marker in liver depended on the presence of HCC in the same organ. This could be explained in two ways: either the ether lipids were secreted by the tumour and taken up by the adjacent residual tissues, or they were synthesized within hepatocytes morphologically unlike cancer cells.

We thank Dr W. K. Chang of the Virus Unit, Medical and Health Department, for the radioimmunoassays of HBsAg. Clara Lai Hing Lee and Frederic Wai provided able technical assistance. This study was supported by a research grant from the University of Hong Kong.

REFERENCES

ANTHONY, P. P., VOGEL, C. L. & BARKER, L. F. (1973) Liver cell dysplasia: a premalignant condition. *J. Clin. Pathol.*, **26**, 217.

BASSENDINE, M. F., CHADWICK, R. G., LYSSIOTIS, T., THOMAS, H. C. & SHERLOCK, S. (1979) Primary liver cell cancer in Britain—a viral aetiology? *Br. Med. J.*, **i**, 166.

BLUMBERG, B. S., LAROUZE, B., THOMAS, W. & 5 others (1975) The relation of infection with the Hepatitis (B) agent to primary hepatic carcinoma. *Am. J. Pathol.*, **81**, 699.

BROWN, R. C., BLANK, M. L., KOSTYU, J. A., OSBURN, P., KILGORE, A. & SNYDER, F. (1975) Analysis of tumour-associated alkyldiacylglycerols and other lipids during radiation-induced thymic leukemogenesis. *Proc. Soc. Exp. Biol. Med.*, **149**, 808.

FRIEDBERG, S. J. & HALPERT, M. (1978) Ehrlich ascites tumour cell surface membranes: An abnormality in ether lipid content. *J. Lipid Res.*, **19**, 57.

HO, J. C. I., WU, P. C. & GIBSON, J. B. (1980) Hepatitis B surface antigen in hepatocytes at necropsy: comparison with serologic results performed post mortem or ante mortem. *Arch. Path. Lab. Med.* (in press).

LAI, C. L., WU, P. C., LAM, K. C. & TODD, D. (1979) Histologic prognostic indicators in hepatocellular carcinoma. *Cancer* (in press).

LIN, H. J., HO, F. C. S. & LEE, C. L. H. (1978) Abnormal distribution of *O*-alkyl groups in the neutral glycerolipids from human hepatocellular carcinomas. *Cancer Res.*, **38**, 946.

LIN, H. J., LIE, M. S. F., LEE, C. L. H. & LEE, D. H. S. (1977) Composition of *O*-alkyl and *O*-alk-l-enyl moieties in the glycerolipids of the human adrenal. *Lipids*, **12**, 620.

NAYAK, N. C., DHAR, A., SACHDEVA, R. & 6 others (1977) Association of human hepatocellular carcinomas and cirrhosis with hepatitis B virus surface and core antigens in the liver. *Int. J. Cancer*, **20**, 643.

OKITA, K., KODAMA, T., HARADA, T. & 6 others (1977) Early lesions and development of primary hepatocellular carcinoma in man: Association with hepatitis B viral infection. *Gastroent. Jpn*, **12**, 51.

PETERS, R. L., AFROUDAKIS, A. P. & TATTER, D. (1977) The changing incidence of association of hepatitis B with hepatocellular carcinoma in California. *Am. J. Clin. Pathol.*, **68**, 1.

SHIKATA, T., YAMAZAKI, S. & UZAWA, T. (1977) Hepatocellular carcinoma and chronic persistent hepatitis. *Acta Path. Jpn*, **27**, 297.

SNYDER, F. & SNYDER, C. (1975) Glycerolipids and cancer. *Progr. Biochem. Pharmacol.*, **10**, 1.

SNYDER, F. & WOOD, R. (1969) Alkyl and Alk-l-enyl ethers of glycerol in lipids from normal and neoplastic human tissues. *Cancer Res.*, **29**, 251.

TONG, M. J., SUN, S. C., SCHAFFER, B. T., CHANG, N. K., LO, K. J. & PETERS, R. L. (1971) Hepatitis-associated antigen and hepatocellular carcinoma in Taiwan. *Ann. Int. Med.*, **75**, 687.

WU, P. C. (1978) Detection of hepatitis B surface antigen in liver biopsies from 655 Chinese patients in Hong Kong. *Asian J. Infect. Dis.*, **2**, 223.

WU, P. C. & LAM, K. C. (1979) Cytoplasmic hepatitis B surface antigen and the ground-glass appearance in hepatocellular carcinoma. *Am. J. Clin. Pathol.*, **71**, 229.